# Molecular genetic testing strategies used in diagnostic flow for hereditary endocrine tumour syndromes

**DOI:** 10.1007/s12020-021-02636-x

**Published:** 2021-02-11

**Authors:** Henriett Butz, Jo Blair, Attila Patócs

**Affiliations:** 1 Department of Molecular Genetics, National Institute of Oncology, Budapest, Hungary; 2 Hereditary Cancers Research Group, Hungarian Academy of Sciences-Semmelweis University, Budapest, Hungary; 3 Department of Laboratory Medicine, Semmelweis University, Budapest, Hungary; 4 Alder Hey Children’s Hospital—NHS Foundation Trust, Liverpool, United Kingdom; 5 Semmelweis University, Budapest, Hungary

**Keywords:** Inherited tumour, Genetic testing, Endocrine tumour syndromes, Next-generation sequencing

## Abstract

**Introduction:**

Although current guidelines prefer the use of targeted testing or small-scale gene panels for identification of genetic susceptibility of hereditary endocrine tumour syndromes, next generation sequencing based strategies have been widely introduced into every day clinical practice. The application of next generation sequencing allows rapid testing of multiple genes in a cost effective manner. Increasing knowledge about these techniques and the demand from health care providers and society, shift the molecular genetic testing towards using high-throughput approaches.

**Purpose:**

In this expert opinion, the authors consider the molecular diagnostic workflow step by step, evaluating options and challenges of gathering family information, pre- and post-test genetic counselling, technical and bioinformatical analysis related issues and difficulties in clinical interpretation focusing on molecular genetic testing of hereditary endocrine tumour syndromes.

**Result and conclusion:**

Considering all these factors, a diagnostic genetic workflow is also proposed for selection of the best approach for testing of patients with hereditary genetic tumour syndromes in order to minimalize difficult interpretation, unwanted patient anxiety, unnecessary medical interventions and cost. There are potential benefits of utilizing high throughput approaches however, important limitations have to be considered and should discussed towards the clinicians and patients.

## Introduction

A joint position paper of the European Reneference Network on rare endocrine diseases (Endo-ERN) was recently published about genetic testing in inherited endocrine disorders [1]. This guideline addressed the opportunities, challenges, limitations and relevance of comprehensive genetic diagnostic testing in rare endocrine conditions in order to achieve an early molecular diagnosis [1]. In this present expert opinion, we summarise the currently valid guidelines relevant for molecular genetic diagnostic workflow used in clinical investigation of hereditary endocrine tumour syndromes. In this group of conditions, genetic counsellors, specialists in endocrinology and oncology must cooperate closely to achieve the best outcome for patients [2].

Generally, whole-exome sequencing results in definitive diagnosis in ~11–40% of the patients depending on disease type [3]. Many rare hereditary cancer syndromes have been identified, however, overall genetic susceptibility is estimated only in 5–10% of all cancer cases [4]. Features that suggest genetic susceptibility are: early age of onset, multiple primary tumours, multifocal sites, bilateral tumour appearance in paired organs, same type of tumour in first or second-degree relatives or same tumour type clustering within a family, rare tumour types (e.g., adrenocortical carcinoma, medullary thyroid cancer, pheochromocytoma), and rare tumours associated with birth defects [5]. In endocrinology, germline mutations are found in the background of diseases at a relatively higher proportion (Table 1) [6–16]. Hence, genetic counselling should be offered for patients and their family members at the presentation of certain tumours (e.g., pheochromocytoma, medullary thyroid, adrenocortical cancer, pancreas neuroendocrine tumours), or if the presentation is at an early age. Patients with endocrine tumour syndromes are under the care of both endocrinologists and oncologists, and optimal treatment requires delivery of care by an endocrine-oncology tumour board. Referral indications for endocrine cancer predisposition assessment are recommended by individual disease guidelines [1, 17–20] but also summarised by the American College of Medical Genetics and Genomics (ACMG) and the national society of genetic counsellors in a practice guideline [21].

**Table 1:** Genetic background of hereditary endocrine tumour syndromes

Endocrine tumour syndromes	Tumours associated with the syndrome [less common ones in parentheses]	Proportion of simplex cases or assumed to be de novo	Proportion of familial cases	Proportion of pathogenic variants detectable	Reference
MEN1	Primary hyperparathyroidism mostly due to hyperplasia, GEP-NET, carcinoid, pitNET, facial angiofibromas, lipomas, collagenomas [adenocortical tumour, bronchial/ thymic NET]	10%	90%	Sequencing identifies a pathogenic mutation in 80–90%—of familial and 65% of simplex cases	[6]
				CNV analysis identifies pathogenic deletion/duplication in 1–4% of cases	
MEN2	Medullary thyroid cancrinoma, pheochromocytoma, primary hyperparathyroidism mostly due to adenoma [mucosal neuromas, intestinal ganglioneuromatosis in MEN2B]	<5%	95%	Sequencing identifies a pathogenic mutation in >95–98% of cases	[7]
HP-JT	Parathyroid adenoma/carcinoma, fibro-ossifying tumours of the maxilla and mandibula, renal and uterine tumours	Unknown		Sequencing identifies a pathogenic mutation in >70%	[8]
				CNV analysis identifies pathogenic deletion/duplication in <30%	
Cowden syndrome	Breast cancer, epithelial thyroid cancer (non-medullary)/adenoma, especially follicular thyroid cancer, endometrial carcinoma, adult Lhermitte-Duclos disease (LDD, cerebellar dysplastic gangliocytoma), trichilemmomas, [hamartomatous intestinal polyps, lipomas, fibromas, genitourinary tumours especially renal cell carcinoma, uterine fibroids]	50–90%	10–50%	Sequencing identifies a pathogenic mutation in 25–80%	[9]
				CNV analysis an identifies pathogenic deletion/duplication (frequency is uncertain)	
MAS	Pituitary adenoma/hyperplasia, Leydig and/or Sertoli cell hyperplasia, ovarian cysts, adrenal hyperplasia	100% (mosaic)	0%	Targeted sequence analysis of lesion biopsy ~80%	[10]
Carney complex	myxomas (cardiac, cutan, mucosal, breast, osteochondromyxoma), adrenal hyperplasia (primary pigmented nodular adrenocortical disease), pituitary adenoma, large-cell calcifying Sertoli cell tumour, thyroid carcinoma, Psammomatous melanotic schwannomas, breast ductal adenoma [thyroid, colon, pancreas, and ovarian carcinoma]	30%	70%	Sequencing identifies a pathogenic mutation in 60%	[11]
				CNV analysis an identifies pathogenic deletion/dupl in 10%	
PJS	gastrointestinal hamartomatous polyps and cancer, breast cancer, cervix (adenoma malignum), endometrium carcinoma, pancreas carcinoma, ovarian sex-cord tumour, testicular Sertoli cell tumour, lung cancer, thyroid nodules/carcinoma	~45%	55%	Sequencing identifies a pathogenic mutation in 81%	[12]
				CNV analysis an identifies pathogenic deletion/duplication in 15%	
PPGL	Pheochromocytoma, paraganglioma [GIST, pulmonal chordoma, renal cell carcinoma, papillary thyroid carcinoma, NET]	Unknown	~40%	Sequencing identifies a pathogenic mutation in 97%—of familial and 30% of simplex cases	[13, 14]
VHL	retinal angioma, spinal or cerebellar hemangioblastoma, adrenal/extra-adrenal pheochromocytoma, clear cell renal cell carcinoma, pancreas NET, endolymphatic sac tumours [multiple papillary cystadenomas of the epididymis or broad ligament]	20%	80%	Sequencing identifies a pathogenic mutation in 89%	[15]
				CNV analysis an identify pathogenic deletion/duplication in 11%	
FIPA					
AIP	Pituitary adenoma	Unknown (rare)	~100%	Sequencing identify a pathogenic mutation in 95%; CNV analysis identify pathogenic del/duplication ~5%	[16]
XLAG	Pituitary adenoma	Most have de novo somatic mosaic genetic alteration	unknown (but rare)	It is caused by duplication of GPR101 gene on chromosome X	

Germline genetic testing influences the proband’s disease on the one hand (actual treatment: e.g., surgical approach, early diagnosis of the potential appearance of multiple tumours by surveillance) and the proband’s family (establishment of the heritability of the disease, family screening/cascade testing and family planning) on the other hand. Therefore, germline genetic testing has an important role in tumour syndromes considering all the relevant legal regulations in force in each country.

## Genetic counselling

The importance of genetic counselling in oncology is highlighted by several guidelines from ACMG, American society of clinical oncology (ASCO) and national comprehensive cancer network (NCCN) ([4, 21], https://www.nccn.org/professionals/physician_gls/default.aspx#detection). For assessment of potential hereditary cancer risk, probands’ medical history and expanded family history have to be investigated.

### Proband history

All information about previous medical history and potential environmental exposition should be gathered including laboratory/histology results, imaging studies, physical and other examination findings and previous medical interventions/treatments focusing on cancer types, cancer site, age of onset and results of potential previous genetic testing.

### Family history

The minimum information required for an adequate cancer family history is defined by ASCO expert statement [4]. Briefly, a three-generation pedigree should be generated indicating all important information relevant to the diseases and potential previously identified familial mutations [4]. The occurrence of bilateral, multiple tumours or tumours characteristic of a certain tumour syndrome affecting different members of the same family requires special attention. Also, clusters of tumours in the family should affect individuals within either the maternal or paternal side [2]. Ethnicity is an important factor influencing the decision to undertake genetic testing as certain populations harbour founder mutations (e.g. *BRCA1/BRCA2* pathogenic variants in the Ashkenazi Jewish population, *SDHD* mutation in Dutch, *VHL* mutation in Germany, Freiburg area, *TP53* mutation in Brasil etc.).

However, most guidelines are based on studies performed predominantly on white populations and may have limited validity in other ethnicities [5, 22].

A genetic test should be offered if—apart from susceptibility based on personal and family history—the following criteria are met: (1) a genetic test is available that has sufficient sensitivity and specificity; (2) the result can be correctly interpreted; (3) a positive result will influence the proband’s diagnosis, therapeutic or cancer risk management; and/or (4) give useful information about cancer risk for family members [2, 5].

The aim of the pre-test counselling is to assess the probability of hereditary cancer predisposition syndrome, to help clinicians develop a differential diagnosis, and offer genetic testing if it is justified. During counselling, it is determined which the most appropriate test is and what information can be expected based on the result. Counsellors explain the benefits and limitations of genetic testing (including possible outcomes) and the possibility of not testing as well. This information helps patients to decide whether or not to have a genetic test and to give informed consent, including signing a consent form [2].

Once a genetic diagnosis is made, counsellors provide recommendations for surveillance, prevention and potential management informed by ref. [5]. They inform patients about personal cancer and recurrence risk, mode of inheritance and give advice about family planning. They also indicate the necessity of additional genetic counselling and genetic testing for at-risk relatives.

Genetic testing performed without appropriate pre- and post-test genetic counselling can result in unnecessary genetic testing, misinterpretation of genetic test results leading to potentially inappropriate medical management (prevention/surveillance/intervention) [23–25]. As human genetic information is sensitive, inheritable and it influences lives of patients and relatives, the absence of genetic counselling can violate ethical standards and result in uninformed decision making about the individuals’ life management [23–25].

Even during genetic counselling, there are limitations in cancer risk assessment. In the family history, there are several factors: small family size, lack of knowledge of family history, deaths in early age, gender imbalance, consanguinity or misattributed parentage that complicate the recognition of inheritance pattern [5, 26, 27]. Apart from family, genetic factors can also challenge the identification of inheritance, such as late-onset tumour manifestation, decreased penetrance and expression, de novo variant, mosaicism and genetic heterogeneity (several genes associated with a certain tumour type) [5, 26, 27]. Due to these limitations, genetic testing is most beneficial when it is performed on the proband affected.

## Genetic testing

Different tests of the same gene have varying levels of sensitivity and specificity. In addition, genetic heterogeneity makes the selection of the best genetic test challenging (Table 2) [28–36].

**Table 2:** Genetic heterogeneity of apparently sporadic endocrine tumours

Organ	Tumour	Potential genetic cause
Pituitary [29]	Adenoma	MEN1, CDKN1B, AIP, PRKAKR1A, GNAS, GPR101, TSC1-2, NF1, SDHx
	Blastoma	DICER1
Thyroid gland [30, 31]	Medullary thyroid cancer	RET
	Non-medullary adenoma/carcinoma	PRKAR1A, PTEN, GNAS, STK11, APC, SLC26A4, WRN
Parathyroid gland [32]	Hyperplasia/adenoma/carcinoma	MEN1, CDKN1B, RET, CDC73, CaSR
Bronchial/gastrointestinal chromaffin cells [33]	Neuroendocrine tumour	MEN1, VHL, NF1, RET, TSC1-2
Adrenal gland [34–36]	Adrenocortical hyperplasia/adenoma/carcinoma	MEN1, PRKAR1A, GNAS, TP53, ARMC5, FH, APC, IGF2 (11p15 imprinting), PDE11A, PDE8B, PRKACA
	pheochromocytoma	RET, VHL, NF1, SDHA, SDHB, SDHC, SDHD, SDHAF2, MAX, TMEM127, KIF1B, EGLN1, HIF2A, FH, PHD1-2, HRAS, ATRX

## Targeted gene testing—pros and cons

For targeted gene testing, conventional Sanger sequencing is the most widely used as a gold standard method [23]. Small regions (~150–800 base pairs) are amplified first by polymerase chain reaction then following a clean-up of the amplification product, sequencing PCR reactions are performed based on dideoxy termination [37]. In the last step, the product of the sequencing reaction is run by capillary electrophoresis where each base is detected by its fluorescent tag after laser excitation. This approach is considered to be a relatively fast and inexpensive strategy if the genomic region of interest is small. Although copy number alterations cannot be assessed it is a very reliable technology for investigating gene sequences base by base. This is a low throughput technique, if long or numerous genes should be tested, it is labour-intensive, time-consuming and costly. Therefore its applicability during the diagnosis phase has been debated. On the other hand, in family screening (or so-called cascade testing) when asymptomatic individuals are tested for an already identified genetic alteration, a targeted genetic test of the characteristic pathogenic variant should be used.

## Next-generation sequencing (NGS)—pros and cons

### Pros

NGS is able to investigate more than 50 genes simultaneously, often at a lower cost than single-gene testing in terms of cost/gene. When NGS multigene panels are planned it is important to include discussion of which genes will be tested during counselling [38]. In addition, when genetic heterogeneity, decreased penetrance and variable expression are likely, or in case of an atypical clinical presentation of a particular cancer syndrome, NGS approaches can save both time and money [39].

### Cons

Although laboratories now offer various cancer susceptibility gene panels, not all genes included in the panels are of unequivocal clinical relevance. Findings identified in genes without clearly established clinical value may be difficult to interpret (see below).

### NGS—technical challenges

NGS comprises of library preparation, sequencing and data analysis (see details in [40]). Each platform has different characteristics regarding reading length, output read a number, cost, and run time [40], and technical standards should meet the recommendations from both the ACMG and European society of human genetics (ESHG) [41, 42]. Repetitive sequences, copy-number variations, long insertion-deletions, structural variants, aneuploidy or epigenetic alterations are usually missed by NGS [23]. In addition, genes having one or multiple pseudo-genes, allele drop-out need special attention. Some of these can also be detected by NGS however, only using additional special steps that have to be clarified by the laboratory performing the test for clear clinical interpretation.

### NGS—bioinformatical challenges

During NGS a huge amount of sequence data is produced that requires special bioinformatics handling and analysis, hence bioinformatics probably represents more challenges compared to the sample analysis [40, 43–45]. Data analysis consists of primary (base calling, read generation), secondary (read alignment and variant calling) and tertiary (variant annotation and interpretation) analysis [43]. Read alignment, variant calling and the depth of sequence coverage influence accuracy significantly [44, 46–49].

### NGS—challenges during variant interpretation

During a WES test 15,000–20,000 variants can be expected [50]. This list has to be narrowed and prioritised to find the one or two disease-causing variant(s). Criteria of interpretation of sequence variants as ‘pathogenic’, ‘likely pathogenic’, ‘variant of uncertain significance’, ‘likely benign’, and ‘benign’ are defined by ACMG in a joint consensus [51]. Based on the classification the term ‘sequence variant’ is recommended in preference to the term ‘mutation’. The ACMG evidence framework for classification uses population data, computational prediction, functional data, segregation, de novo occurrence, allelic data (please find it detailed in [51]). The assessment of co-segregation of a certain variant with a phenotype (tumour occurrence) should be done by the clinical geneticist. This adds further evidence for the classification of variants, however incomplete penetrance, late-onset disease presentation, or variable phenotype can lead to lack of segregation [51]. So-called trio-sequencing (affected proband and both parents) is a useful tool in interpretation [52] as it helps to exclude heterozygous rare variants based on the genotype of the parents [52].

It is possible that a pathogenic variant is not identified in an affected individual. Apart from technical problems, the reasons for this include a pathogenic variant in another gene that was not tested or in a gene not yet identified, phenocopy and additionally, sporadic occurrence. In these cases, affected probands and first-degree relatives can be kept under clinical surveillance.

To estimate the relevance of variants of uncertain significance (VUS) is challenging [48, 51, 53]. The functional effect of a VUS can be characterised by RNA testing (splice effect), loss of heterozygosity testing in tumour tissue and by investigating healthy first-degree relatives (primarily parents) [51].

Due to constantly increasing data and knowledge, reevaluation and reanalysis of genetic test results are possible. Moreover, laboratories should have a protocol regarding variant-level and case-level reevaluation that is supported by ACMG guidelines [54].

Laboratories should also have a communicated policy about reporting incidental (secondary) findings [55] guided by ACMG recommendations (including a minimum list of 59 medically actionable genes for reporting) [55].

Screening for hereditary cancer syndromes is only recommended in high-risk patients due to higher VUS rate, the equivocal clinical value of variants with low-penetrance or newly discovered genes and secondary findings [56].

## Cautions for high throughput testing, which platform to choose

As noted above, handling large data sets generated by high-throughput technologies can result in high numbers of false-positive results and incidental findings. This risk correlates with the number of genes tested simultaneously. As a consequence overtesting, overdiagnosis, and overtreatment were described as major side effects of high-throughput approaches [57].

Whole-genome sequencing (WGS) covers the whole coding and noncoding regions of the genome, and for this reason, it may be the preferred genetic test. However, among NGS approaches it gives the least average coverage and it is the most expensive technology. In addition, the interpretation of noncoding variants and VUSs results in the most uncertainty. Whole-exome sequencing (WES) covers all protein-coding regions of the human genome. Although this comprises ~1–2% of the genome, it includes approximately 85% of known disease-causing mutations, making it a more feasible option [58]. It can provide an average exome coverage of 90–95% due to sequence complexity, and due to the uneven depth of coverage, its sensitivity is usually lower than targeted disease panels. Targeted gene panels by NGS are therefore the most widely used approach in clinical practice [42]. By focusing on a smaller, limited set of genes it provides higher analytical sensitivity. Because the role of genes included in these panels are known to be associated with a particular condition the detection rate (positive finding) is also higher compared to WES [23]. However, it should be mentioned that for various commercially available services the gene panels are ‘virtual’ because WES is performed and, based on clinical data, virtual panels are evaluated.

Consequently, based on the guideline recommended by the ESHG ‘*for diagnostic purpose, only genes with a known* (*i.e., published and confirmed) relationship between the aberrant genotype and the pathology should be included in the analysis*’ and ‘*for the sake of comparison, to avoid irresponsible testing, for the benefit of the patients,* ‘*core disease gene lists*’ *should be established by the clinical and laboratory experts*’ [42]. The addition of low penetrance or newly identified genes to diagnostic targeted panels without specific actionability is unethical and can be problematic for interpretation [2, 42]. These tests should be performed in research settings and patients have to be correctly informed in advance [42].

Therefore, although high-throughput sequencing (WGS, WES, ‘experimental’ panels) are technically available and have many advantages they still represent diagnostic challenges that have to be considered in clinical practice [53, 59].

## Clinical relevance of genetic testing in hereditary endocrine tumour syndromes

The clinical value (diagnosis, tumour risk assessment, therapy, surveillance, family screening/planning) of genetic testing differs among endocrine tumour syndromes, hence disease-specific guidelines have to be followed.

Establishing a diagnosis is usually based on clinical criteria in a proband, and confirmed by the result of the genetic test. Also, in atypical cases (for example due to decreased penetrance or variable expression) genetic testing can be an important part of the diagnosis. However, genetic testing in asymptomatic family members (so-called cascade testing), establishes an early diagnosis which may have significant consequences.

While genetic tests in nearly all endocrine tumour syndromes contribute to tumour risk assessment, overall, therapeutic decisions in endocrine tumour syndromes are rarely based on germline genetics. Therapy includes chemo-, hormone, targeted- radio-, radionuclide therapy and surgical interventions (both preventive and therapeutical). Targeted oncological therapy—if used in individual cases—is rather based on somatic alterations. However, germline variants can influence surgical approaches and timing for specific surgeries. For instance, in a patient with renal cell carcinoma when a pathogenic germline *VHL* variant is confirmed surgeons prefer nephron-sparing surgery over total nephrectomy in order to spare organ function as long as possible since tumour re-occurrence in the same or on the other side is more likely compared to sporadic cases [60].

When genotype-phenotype correlation is strong, surveillance and preventive measures can be based on genetic findings. In MEN2 syndrome, the timing of prophylactic thyroidectomy is based on the particular *RET* mutation [61]. On the other hand, in MEN1 syndrome, there is no clear genotype-phenotype correlation, hence no individualised mutation-dependent surveillance is present. The actual clinical practice guideline for MEN1 syndrome recommends a comprehensive surveillance scheme starting at the age of 5 years in order to early detect and manage MEN1-associated tumours [62]. Starting age is usually determined by the earliest case reported with each manifestation, however, the sensitivity of some of the surveillance tests are questionable and their application has not been proven to be effective in early diagnosis or long term prognosis [35, 63].

Regarding family screening or so-called cascade testing, knowing the pathogenic variant characteristic for a family, targeted genetic tests provide a simple and cheap method for cascade testing. Family screening has to be voluntary no one can be forced against his/her will. Advantages and disadvantages have to be discussed during genetic counselling. However, cascade testing has an indisputable role in prevention. Involving asymptomatic carriers in relevant surveillance protocols helps the early diagnosis and favours early medical interventions. Also, it gives the opportunity to decide about family planning. Each person has the right to make informed decisions regarding his/her life management in the light of potential carrier status of a pathogenic germline variant [23, 24]. The mode of inheritance, the onset of the disease, outcome/prognosis, therapeutic and preventive measures all influence the decisions individuals make with regards to family planning and should be discussed during genetic counselling [64]. For carriers or probands with autosomal dominant tumour syndromes options for prenatal (by chorionic villus sampling and amniocentesis) and preimplantational genetic testing (by assisted reproduction) should be also discussed following regulations available in different countries [65].

To assess the clinical relevance of a VUS is challenging [66]. This can be further complicated because reporting of genetic test results may differ among laboratories in spite of the relevant guidelines [51, 66]. Regular re-classification can be asked or should be provided by the laboratories [51, 66]. Variant-phenotype segregation in the family assessed by the clinical geneticist can help to estimate the clinical relevance [51]. As not all VUS are equal regarding risk estimation, the ACGS Best Practice Guidelines for Variant Classification in Rare Disease can also be used [67, 68]. Therefore, genetic counsellors have to individually decide in each case about the clinical relevance associating with a VUS. According to the IARC classification system [68, 69], VUS should not be used for predictive testing in at-risk individuals and the surveillance should be based on family history. Moreover, the therapeutical decision is not recommended to be chosen based on a VUS finding [70].

The genetic test is only one criterion in the establishment of the diagnosis of tumour syndromes. In some cases, a patient despite having a clear phenotype characteristic for an endocrine tumour syndrome the genetic test fails to identify the pathogenic alteration. This phenomenon is called a phenocopy. Of endocrine tumour syndromes, it frequently occurs in relation to MEN1 [71]. If the clinical diagnosis—based on phenotype—is clear then the patient should be treated accordingly. These patients and their family members at risk should also participate in the recommended surveillance programs following the relevant guideline of the particular tumour syndrome.

## Proposed diagnostic workflow in endocrine tumour syndromes

As we described above, the more genes we test the more uncertainty we have to deal with. Selection of the proper test is the task of genetic counsellors after patients are referred to genetic consultation by clinicians (endocrinologist, oncologist).

**Fig. 1:**
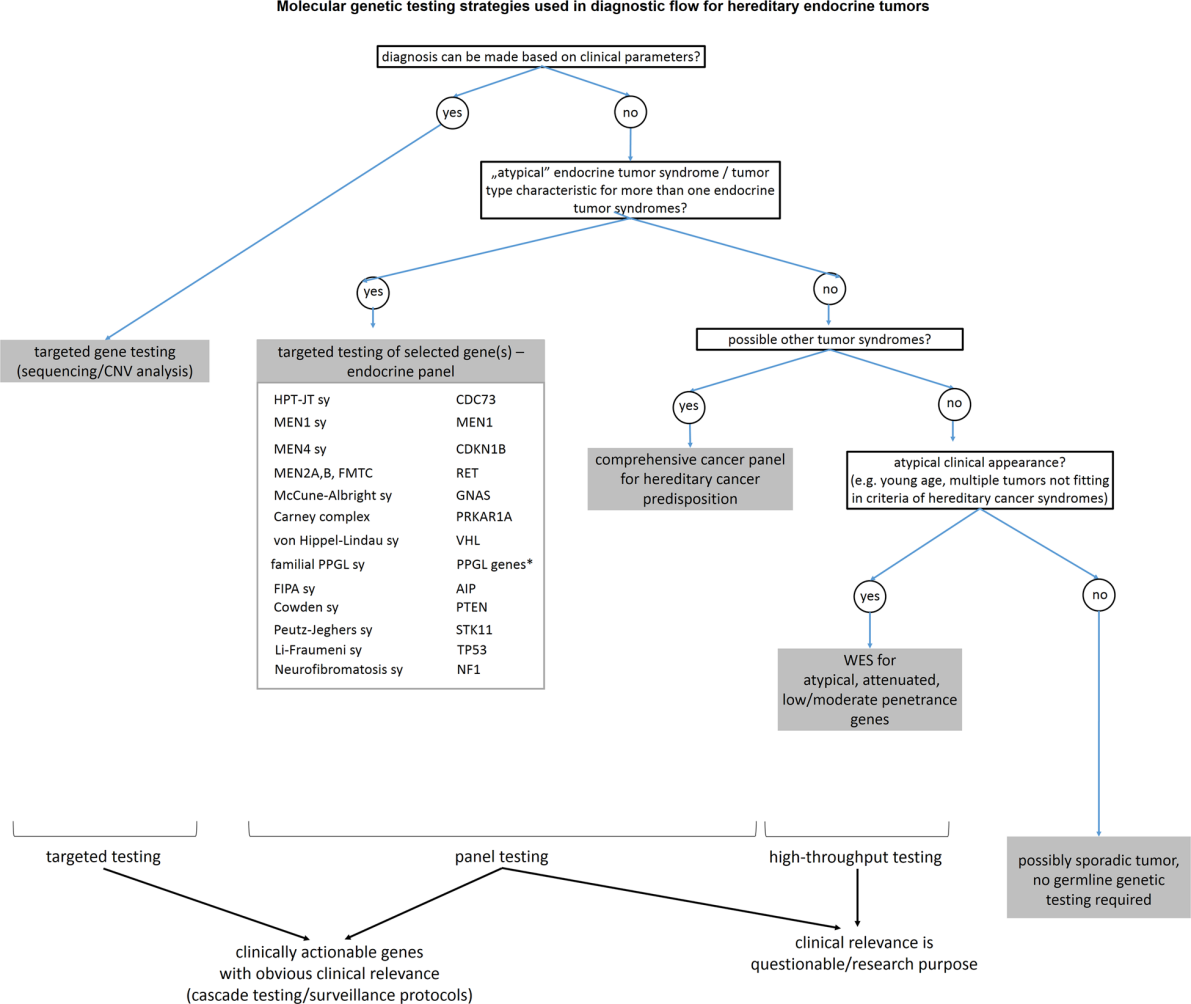
Proposed workflow of molecular genetic testing of endocrine tumour syndromes. *hereditary PPGL genes, see details in the text

Whenever it is possible (e.g. typical clinical presentation) targeted gene testing (usually by conventional Sanger sequencing and/or MLPA for detection of copy number alteration) is suggested (Fig. 1). Targeted tests are the fastest and the most cost-efficient option. However, when genetic heterogeneity can be raised (one tumour can be part of several tumour syndromes, Table 1) or more than a few genes can be in the background (e.g., in paraganglioma-pheochromocytoma syndrome) disease-specific/endocrine (targeted) gene panel testing can be recommended with all the advantages listed above [2]. Endocrine tumour syndromes are quite well defined, however, there are overlaps with other tumour syndromes, where endocrine tumours are not obligatory but can be part of the syndromes (e.g., Li-Fraumeni syndrome, familial adenomatous polyposis etc.). Therefore, when other tumour syndromes are possible, or clinical features are atypical comprehensive cancer panel can be offered (being aware of potential uncertain and secondary findings—e.g., a VUS in *TP53* gene—that can complicate the picture) [2, 72, 73]. If no pathogenic variant is identified but the clinical suspicion is still strong, in atypical cases, or in case of seeking potential low/moderate penetrance genes WES could be performed keeping in mind detection rates and all uncertainties listed above.

## Conclusions

In the last decade, NGS based strategies have been revolutionised genetic testing and high-throughput techniques are widely introduced into every day clinical practice. The application of NGS allows rapid and cost-effective testing of multiple genes. Therefore, an increased demand from health care providers and society has been pressing to shift genetic tests towards high-throughput approaches. Besides all advantages, however, the application of NGS is associated with increased uncertainty in result interpretation. Challenges in technical, bioinformatical processes and in interpretation presented in this work highlight the increased uncertainty of the clinical value of the results. Therefore, following ACMG and ESHG guidelines, it is still recommended phenotype-based targeted testing and to use as small-scale approach as possible in order to minimalize difficult interpretation (e.g., VUS), unwanted patient anxiety, unnecessary medical interventions and cost. Selecting the most suitable genetic test is the part of the work of clinical geneticists. Fortunately, endocrine tumour syndromes are quite well defined, and only selected cases require WES. Still, genetic heterogeneity, decreased penetrance, variable expression or mosaicism can challenge diagnostic workflow. As the result of germline genetic test influences the life of the proband, including treatment and surveillance and the life of his–her family through family screening/cascade testing and family planning, germline genetic testing has an important role in the management of endocrine tumour syndromes.
